# Formulation and evaluation of simvastatin cubosomal nanoparticles for assessing its wound healing effect

**DOI:** 10.1038/s41598-023-44304-2

**Published:** 2023-10-20

**Authors:** Lamiaa M. Ahmed, Khaled M. A. Hassanein, Fergany A. Mohamed, Tahani H. Elfaham

**Affiliations:** 1 Department of Pharmaceutics, Faculty of Pharmacy, Assiut University, Assiut, 71526 Egypt; 2 Department of Pathology and Clinical Pathology, Faculty of Veterinary Medicine, Assiut University, Assiut, 71526 Egypt

**Keywords:** Drug discovery, Nanoscience and technology

## Abstract

Wound healing is one of the most challenging medical circumstances for patients. Pathogens can infect wounds, resulting in tissue damage, inflammation, and disruption of the healing process. Simvastatin was investigated recently, as a wound healing agent that may supersede the present therapies for wounds. Our goal in this paper is to focus on formulation of simvastatin cubosomes for topical delivery, as a potential approach to improve simvastatin skin permeation. By this technique its wound healing effect could be improved. Cubosomes were prepared using the top-down method and the prepared cubosomes were characterized by several techniques. The most optimal simvastatin cubosomal formulation was then included in a cubogel dosage form using different gelling agents. The results showed that the average particle size of the prepared cubosomes was 113.90 ± 0.58 nm, the entrapment efficiency was 93.95 ± 0.49% and a sustained simvastatin release was achieved. The optimized formula of simvastatin cubogel displayed pseudoplastic rheological behavior. This same formula achieved enhancement in drug permeation through excised rat skin compared to free simvastatin hydrogel with flux values of 46.18 ± 2.12 mcg cm^−2^ h^−1^ and 25.92 ± 3.45 mcg cm^−2^ h^−1^ respectively. Based on the in-vivo rat studies results, this study proved a promising potential of simvastatin cubosomes as wound healing remedy.

## Introduction

The skin serves as a barrier and protector against alien invaders. As a result, maintaining skin integrity is critical to avoid serious issues. One of the most challenging health situations for some patients is wound healing^1,2^. Wound healing is a four stages process; haemostasis, inflammatory response, proliferative stage and tissue remodelling^3^. Numerous pharmaceuticals and herbal medicines have reportedly been shown to aid in wound healing^4^. Simvastatin (Sim) is a member of statins which are effective for treating hyperlipidemia through competitive inhibition of 3-hydroxy-3-methylglutaryl co-enzyme-A HMG-CoA) reductase, decreasing cholesterol biosynthesis. Other than its anti-hyperlipidemic activity, recent research has shown that Sim has immunomodulatory, anti-inflammatory, and angiogenic properties and may also speed up the healing of wounds^5,6^. In previous investigations^7–10^, Sim has been demonstrated to have a critical part in the development of the epithelium; via reducing epidermal farnesyl pyrophosphate (FPP) levels and elevating keratinocyte emigration. Additionally, Sim causes variety of cells to secrete vascular endothelial growth factor (VEGF), which increased angiogenesis. So Sim is regarded as an innovative treatment approach for the recovery of wounds. However, Sim has a number of drawbacks, such as poor water solubility, which makes it poorly absorbed; only about 5% of an oral dose reaches the circulatory system^11^. This drawback can be resolved by incorporating Sim into a suitable nanocarrier for topical delivery, which will also increase the efficacy of wound healing. Several attempts have been made in this direction such as preparation and evaluation of Sim loaded provesicular elastic system which showed a considerable increase in the expression of vascular endothelial growth factor and collagen type-I compared to the free medication so wound healing was improved^1^. Örgül, et al., proved the role of nanostructured lipid carriers (NLCs) in enhancing Sim healing effect on wounds^12^. Also, topical nanosponge were successfully studied to increase penetrability and passive accumulation of incorporated drug in the skin^13^. Transdermal administration of sertraline using transfersomes as vesicular carriers was developed and described to increase its skin permeation. The findings demonstrated that transferosomes are promising carriers for improving medication administration through the skin^14^.

Cubosomes are nanostructured liquid crystalline particles (LCNPs) with superior characteristics like thermodynamic stability, bioadhesion, a large surface area, the capacity to incorporate amphiphilic, hydrophobic and hydrophilic drugs, and  a controlled release scenario is also possible with these systems. The inner cubosomal^’^ structure is comparable to that of stratum corneum, so cubosomes promote medication entrance over the skin's epidermis, making them appealing drug delivery system for topical use^15,16^.

Cubosomes are three-dimensional honeycomb structures made of lipid bilayers that are bicontinuous curved, separating two internal watery channels. They act as a nanocarriers for several substances, including medicines, peptides, and proteins^17^. This study aimed to formulate simvastatin cubosomes for topical delivery and providing accurate evaluation data on the wound healing effects of simvastatin.

## Materials and methodology

### Materials: font and location

Simvastatin (Sim) was a gift from PHARCO Pharmaceuticals Inc. (Alexandria, Egypt), Glyceryl monooleate(GMO) and poloxamer_407_ (F_127_) was purchased from sigma-Aldrish. St. Louis, MO, USA, chitosan (HMW) was obtained from alpha chemika Co., Cairo, Egypt. labrasol®(Caprylocaproylpolyoxyl-8glycerides) supplied by Gattefosse CO. (Saint Priest, France). All other compounds were of analytical grade and utilized as received.

### Methodology

#### Preparation of cubosomes

Glyceryl monooleate (GMO)/poloxamer_407_ bulk cubic gel was fragmented to create cubic nanoparticles using top-down method. GMO and poloxamer_407_ (F_127_) were melted over a water bath at 60 °C, followed by adding Sim with stirring till complete dissolving. 0.25 ml of deionized water, which was gradually added with vortex mixing until homogenous state was achieved. After equilibration for (24–48 h) at ambient temperature, a cubic phase gel that is optically isotropic was generated. To disrupt the cubic gel, 10 ml of deionized water were introduced with magnetic stirring. Intermittent probe sonicator was used to fragment the coarse dispersion at 20 °C for 15 min (Pulse 5 on, 2 off), and then was homogenized by passing specified number of cycles through high pressure homogenizer to obtain cubosomal nanoparticles. The nanoparticles were then stored in a refrigerator (4–8 °C) until required. The composition of prepared cubosomal formulations is presented in Table 1.

**Table 1:** Percents of components of the prepared formulae.

Formulation code	GMO %(w/v)	Poloxamer_407_% (w/v)	Drug % (w/v)	Sonication time (min)	Homogenization speed (cycles)
F1	2	0.5	0.25	15	2
F2	4	0.5	0.25	15	2
F3	6	0.5	0.25	15	2
F4	2	1	0.25	15	2
F5	4	1	0.25	15	2
F6	6	1	0.25	15	2
F7	2	1.5	0.25	15	2
F8	4	1.5	0.25	15	2
F9	6	1.5	0.25	15	2
F10	2	0.5	0.5	15	2
F11	2	0.5	0.35	15	2
F12	2	0.5	0.15	15	2
F13	2	0.5	0.25	10	2
F14	2	0.5	0.25	20	2
F15	2	0.5	0.25	15	1
F16	2	0.5	0.25	15	3

#### Characterization of prepared cubosomal formulations

##### Determination of particle size, polydispersity index and zeta potential

Malvern Zetasizer Nano Series-based dynamic light scattering (DLS) technology was used to determine these parameters, after 100 fold dilution of formulations with distilled water as reported by Eldeeb et al.^18^

##### Determination of entrapment efficiency and drug loading

The un-entrapped drug was seprated from LCNPs dispersion using the dialysis bag method. Where the prepared formulations placed and dialyzed against 100 ml of distilled water for 3 h at 200–300 rpm. Then, the dialysis bag content was analyzed spectrophotometrically at 238 nm after dilution with methanol against blank solution. It was a modified method of that reported by Swarnakar et al.^19,20^

$${\text{Entrapment efficiency }}\left( {{\text{EE}}\% } \right) = \user2{ }\frac{{\text{actual amount of drug in nanoparticles }}}{{\text{theoritical amount of drug in nanoparticles }}} \times 100$$

$${\text{Drug loading }}\left( {{\text{DL}}\% } \right) = \frac{{\text{weight of recoverd drug }}}{{\text{ Total weight of SIM cubic nanoparticles }}} \times 100$$

##### Transmission electron microscopy (TEM)

The samples were inspected by putting a 5-µl volume of the prepared cubosomes suspension onto carbon-coated copper grid, and letting the cubosomes settle for 3–5 min. Then, the excess fluid was removed by wicking it off with an absorbent paper and imaged digitally using a Gatan axis-mount 2 k 2 k digital camera after being adversely stained in 2% uranyl acetate for 3–5 min^21^.

##### In-vitro release study

Sim^’^release from cubic nanoparticles was evaluated over 48 h period, using dialysis bag method (MWCO 12,000–14,000 Da, Spectrum Labs Inc., USA). After separation of free drug from cubosomes, the rate of drug release was evaluated by adding accurately measured volume of each preparation equivalent to 3.5 mg of Sim) within the donor area of the experimental unit which was assembled as a dialysis bag. The release medium (100 ml phosphate buffer, pH 7.4 and sink condition was maintained using 0.25% (w/v) of sodium lauryl sulfate (SLS)) which included into the receptor compartment. The experiment was carried out in a water bath that was shaken under thermostatic control at 70 rpm and a temperature of 37 ± 0.5 °C. Samples were taken out of the receptor compartment (5 ml) and replaced with the same volume of fresh release medium at regular intervals. Spectrophotometric analysis was performed on all samples at λmax of 238 nm, each experiment was made in triplicate. Under the same sink conditions, the release rates of several formulations were compared to those of the drug suspension^22,23^.

#### Formulation and characterization of cubogels

##### Preparation of simvastatin loaded cubosomal gels (cubogels)

The optimized cubosomal formulation was selected to be incorporated within different gelling agents (high molecular weight (HMW) chitosan, carbopol _934_ and hydroxypropyl methyl cellulose (HPMC)). HPMC based cubogels were prepared by sprinkling (4% w/v (G7)—5% w/v (G8)—6% w/v (G9)) of HPMC over certain amount of cubosomal dispersion under magnetic stirring till hazy dispersion was formed, followed by refrigeration overnight^24^. For preparation of chitosan based cubogels and carbopol based cubogels, see supplementary data. Different solubilizers were used in the preparation of cubogels as transcutol (Trans), labrasol (Lab) and propylene glycol (PG)^25^. The composition of the prepared cubogels is shown in the supplementary data.

##### Determination of rheological characteristics of the investigated cubogels

Brookfield Programmable Rheometer was used to determine rheological behavior of the prepared cubogels. Each sample (5 g) was placed in a vial where T–F,96 spindle was incorporated and was allowed to move with different speeds (10–100 rpm), every measurement was performed in triple.

##### Drug release from different cubogels

The same procedure adopted for studying drug release from cubosomes with a specified amount of cubogel (1.5 g) containing (3.75 mg) of drug were applied using the dialysis method.

##### Ex-vivo permeation study

The permeability of Sim was assessd through rat hairless skin using samples (1.5 g) of each cubogel. Male Wister rats with average weight of 200–250 g were used and the experimental procedure was according to the institutional animal ethical committee of faculty of pharmacy, Assiut university (Approval no: S24-21). The rats were sacrificed by neck dislocation, before the trial began and a full-thickness layer of skin was removed. The dorsal area was rinsed with water then phosphate buffer (pH 7.4)^26^. Using a two-open ended tubes, the rat skin was attached to one end where stratum corneum side faced the donor compartment and the another side was toward the receptor compartment^27^. The experiment was carried out similarly to that of drug release. Sim content was determined by analyzing the samples in UV region at λmax 238 nm against a similarly treated blank.

#### Evaluation of in-vivo wound healing activity

One week prior to the start of the trial, twelve male adult Wistar rats weighing 200–250 g were chosen randomly from the animal house, any rats out of these characterizations were excluded, each rat was housed in a cage for acclimatization. They had access to standard chew pellets and water. The experimental procedure was performed in compliance with the institutional animal ethical committee of faculty of pharmacy, Assiut university (Approval no: S24-21) and followed the guide for the care and use of laboratory animals,8^th^ edition, National Academies Press, Washington, DC^28^.

##### Experimental design

The animals were divided randomly into 4 groups (n = 3). All animas were sedated with a mixture of 50 mg/kg ketamine and 5 mg/kg midazolam before shaving the dorsal hair of each animal with hair depilatory cream. Using a round template, animals' dorsal skin was punctured to obtain 1-cm-diameter circular wound. Four different animals treatments were performed. Group 1 containing 3 healthy rats acted as (-ve control), group 2 containing 3 wound induced rats and received normal saline (+ ve control) to observe the spontaneous wound healing process of animals in the absence of any treatment, group 3 containing 3 wounded rats treated with Sim loaded hydrogel, to elucidate the importance of Sim being included into cubosomes and group 4 which have 3 rats each with two wounds; one was received blank cubosomal gel and the other was treated with selected Sim loaded cubosomal gel to assess Sim’s performance as a wound healing agent. Treatments were coded and applied by an assistant and kept masked for the researcher to reduce any unintentional biases. All treatments were applied once daily over 14 days^29,30^.

##### Wound closure

At intervals of 4, 7, 11, and 14 days after the trial's start, the reduction in wound area was monitored and measured using a digital caliber to evaluate wound healing. The following equation was used to compute the gradual decrease in the wound area:

$$\% \, {{of}} \, {{wound}} \, {{reduction}} \, = \, \left( {{{A}}_{{{0}}} - \, {{A}}_{{{t}}} / \, {{A}}_{{{0}}} } \right) \, \times \, {{1000}}$$

where A_0_ implies the wound area at zero time and A_t_ denotes the wound area at specific time^1^.

##### Histopathological study

On the 14th day, rats were sacrificed by neck dislocation, and sections of each group's dorsal tissue were removed. Tissue samples from dorsal skin were preserved in 10% neutral buffered formalin. Then dehydration by ascending grades of alcohol, clearing by xylene and embedding in paraffin was made. Finally, hematoxylin and eosin (H&E) dyes stained the 4–5 microns thick samples to look at the tissue microarchitecture^31^.

### Ethics declarations

1. The experimental procedures were performed in compliance with the institutional animal ethical committee of faculty of pharmacy, Assiut university (Approval no: S24-21). This committee is a branch of Assiut University Research Ethics Committee (AUREC) which is a licensed approved committee. The ordinary processes followed in such situations, the candidate represents his request to perform animals or humans studies to the local faculty ethical committee. His request is accompanied with a copy of the initial study protocol sheet, showing clearly the intended work steps on the animals or humans and the purpose of the study and the importance of including this part in the study. The committee assignes a date to orally discuss the matter with the candidate and ask any questions to inform them clearly. Then the approval sheet was delivered to the candidate .
2. All methods were carried out in accordance with official guide of Assiut University Research Ethics Committee (AUREC), chapter-4. (included in the related files)
3. We had reviewed also the ARRIVE guidelines, All methods were carried out in accordance with the 10 comments of ARRIVE guidelines concerning engagement of animals in research studies.

### Statistical analysis

For elucidation of significance between the different groups, both one-way analysis of variance (ANOVA) and the newman-Keuls post-hoc test were applied (* (*p* < 0.05), **(*p* < 0.01), (*** (*p* < 0.001), NS (non-significant). For all expermints, each one was performed in triplicates and the results were presented as mean ± SD. Pearson correlation coefficient was calculated according to the equation explained by Wu, W. J., & Xu, Y^32^, to describe the correlative degree of two variables of fixed distance.

## Results and discussion

### Preparation of cubosomes

Preliminary trials were conducted to determine the optimal concentration of GMO and F_127_ with optimal sonication and homogenization conditions (Table 1). Sixteen formulae were prepared as shown previously, Table 1.

### Characterization of Sim loaded cubosomes

#### Particle size, PDI and zeta potential

Sim loaded cubosomes were effectively prepared as an aqueous dispersion with mean particle size from 81.67 ± 2.68 nm (F7) to 184.75 ± 3.61 nm (F6) (Table 2). Concentrations of GMO and F_127_ have significant effect on average particle size as shown in Tables 1, 2. Particle size was increased significantly (^**^p < 0.01) with increasing GMO from 113.90 ± 0.58 nm (F1) to 183.50 ± 21.24 nm (F3) due to increasing viscosity, that may oppose emulsification of cubosomes^33^. On the other hand, increase in F_127_ concentration led to smaller (^***^p < 0.001) nanoparticles formation, as F1 (0.5% F_127_) was with particle size 113.90 ± 0.58 nm while that for F7 (1.5% F_127_) was 81.67 ± 2.68 nm. Which could be attributed to the reduction in surface tension during emulsification with higher amounts of F_127_^18^.

**Table 2:** Results of characterization parameters of simvastatin loaded cubic nanoparticles.

Formulation number	Particle size (nm)	PDI	Zeta potential	EE %	DL %
F1	113.90 ± 0.58	0.14 ± 0.01	− 21.80 ± 4.25	93.95 ± 0.49	8.57 ± 0.04
F2	145.87 ± 0.63	0.21 ± 0.00	− 23.40 ± 2.66	75.20 ± 0.42	3.99 ± 0.01
F3	183.50 ± 21.24	0.33 ± 0.08	− 24.20 ± 1.15	60.50 ± 1.41	2.25 ± 0.07
F4	090.80 ± 1.10	0.24 ± 0.01	− 10.40 ± 2.37	84.40 ± 1.55	6.57 ± 0.11
F5	108.85 ± 1.34	0.20 ± 0.01	− 14.65 ± 2.05	84.00 ± 1.41	4.03 ± 0.06
F6	184.75 ± 3.61	0.39 ± 0.00	− 18.20 ± 0.42	80.75 ± 1.62	2.78 ± 0.03
F7	081.67 ± 2.68	0.26 ± 0.01	− 08.20 ± 0.42	74.25 ± 0.92	5.03 ± 0.05
F8	093.48 ± 1.16	0.24 ± 0.01	− 15.55 ± 0.35	89.30 ± 2.40	3.89 ± 0.12
F9	142.75 ± 33.16	0.30 ± 0.09	− 12.85 ± 2.19	88.48 ± 1.15	2.86 ± 0.04
F10	–	–	–	–	–
F11	111.82 ± 0.11	0.15 ± 0.03	− 22.40 ± 0.54	81.80 ± 1.50	10.20 ± 0.17
F12	112.40 ± 0.45	0.18 ± 0.01	− 23.80 ± 2.12	86.60 ± 1.97	4.93 ± 0.11
F13	116.55 ± 7.74	0.18 ± 0.03	− 19.40 ± 1.58	81.00 ± 2.61	7.53 ± 0.22
F14	111.70 ± 2.74	0.16 ± 0.01	− 21.05 ± 3.30	78.00 ± 1.67	7.30 ± 0.14
F15	170.00 ± 3.54	0.35 ± 0.02	− 23.65 ± 3.60	84.30 ± 1.73	8.27 ± 0.50
F16	150.10 ± 42.28	0.25 ± 0.10	− 26.10 ± 2.26	87.66 ± 5.69	8.05 ± 0.48

Upon increasing homogenization cycles from 1cycle (F15) to 2cycles (F1), significant decrease (^*^p < 0.05) in particle size was observed from 170.00 ± 3.54 nm to 113.90 ± 0.58 nm respectively (Tables 1, 2), due to higher mechanical shear^34^. While further increase to 3 cycles (F16) resulted in increase of particle size to 150.10 ± 42.28 nm, that may be attributed to aggregation of fine particles so producing bigger ones.

Slight reduction in average particle size was obtained upon increasing sonication time (10 min (F13),15 min (F1) and 20 min (F14)) (Tables 1, 2). However, there was no significant effect of tested drug concentrations (0.15% (F12), 0.25% (F1), 0.35% (F11) on average particle size (Tables 1, 2).

The values of PDI obtained were between 0.16 ± 0.01 (F14) and 0.39 ± 0.00 (F6) (Table 2), which indicated acceptable size distribution of cubosomal nanoparticles.

The results of zeta potential were obtained with high negative values for most formulations (Table 2). This may be caused by ionization of free oleic acid in GMO and adsorbed onto the cubosomes' surface^35,36^. The formula (F10) was not completed due to precipitation of the drug so it was not included in the characterizations.

#### Entrapment efficiency and drug loading

Entrapment efficiency of Sim in cubic nanoparticles is directly affected by increasing GMO concentrations (Tables 1, 2). Significant increase (^**^p < 0.01) in EE% upon increase GMO from 74.25 ± 0.92% (F7) to 88.48 ± 1.15% (F9) was observed. These results were attributed to Sim’high lipophilicity, so higher GMO resulted in more space for drug incorporation^37^. But this effect was reversed with lower concentration of F_127_ (0.5%) as increase GMO level resulted in significant decrease (^***^p < 0.001) in EE% from 93.95 ± 0.49% (F1) to 60.50 ± 1.41% (F3) (Tables 1, 2). Also DL% reduced from 8.57 ± 0.04% to 2.25 ± 0.07% for the same formulations that could be explained by insufficient amount of polymer required to stabilize cubosomes structure^38^.

EE% and DL% were also affected by polymer concentrations as increasing F_127_ concentrations resulted in significant decrease (***p < 0.001) in entrapment from 93.95 ± 0.49% (F1) to 74.25 ± 0.92% (F7), and loading values were reduced from 8.57 ± 0.04% to 5.03 ± 0.05% for the same formulations (Tables 1, 2). This attributed to the polymer's solubilizing activity, which caused the partitioning of a lipophilic drug into the aqueous phase during the change from the cubic gel phase to cubosomes^39^. But solubilization effect of polymer is negligible at high GMO content (4% and 6%), as with increasing F_127_ concentrations significant increase (^***^p < 0.001) in EE% was observed from 60.50 ± 1.41% (F3) to 80.75 ± 1.62% (F6) and 88.48 ± 1.15% (F9). Also, DL% was increased significantly (^**^p < 0.01) from 2.25 ± 0.07% (F3) to 2.78 ± 0.03% (F6) and 2.86 ± 0.04% (F9) (Tables 1, 2). These results can be justified to be due to the more predominant effect of lipids.

With increasing drug concentration, there were significant increase (^**^p < 0.01) in EE% and DL%; as observed with F12 (86.60 ± 1.97%, 4.93 ± 0.11%) and F1(93.95 ± 0.49%, 8.57 ± 0.04%), while with further increase in drug level to 0.35% the reverse effect was obtained significantly (^*^p < 0.05) between F11 (81.80 ± 1.50%) and F1 (Tables 1, 2) and precipitation was noticed with drug concentration 0.5% (F10), This was caused by excessive drug levels disrupting the cubic phase's consistency^40^.

Application of high shear energy for longer time (20 min) resulted in significant reduction (^**^p < 0.01) in entrapment % from 93.95 ± 0.49% (F1) to 78.00 ± 1.67% (F14) and loading % from 8.57 ± 0.04% to 7.30 ± 0.14% (Tables 1, 2). This was caused by drug expulsion or diffusion upon exposure to higher shear^41^. While application of 10 min resulted also in significant reduction (^**^p < 0.01) in entrapment and loading values, which was observed with F1 and F13 (Tables 1, 2), that can be regarded to shorter time for stabilization of the nano-system. So it could be concluded that application of probe sonicator for 15 min was the optimum time.

Application of different homogenization speeds; 1cycle (F15), 2 cycle (F1) and 3 cycles (F16) was appeared has no statistical significance on entrapment efficiency and drug loading (Table 2) which in concordance with Lai, J., et al.^42^

#### Transmission electron microscopy (TEM)

**Figure 1:**
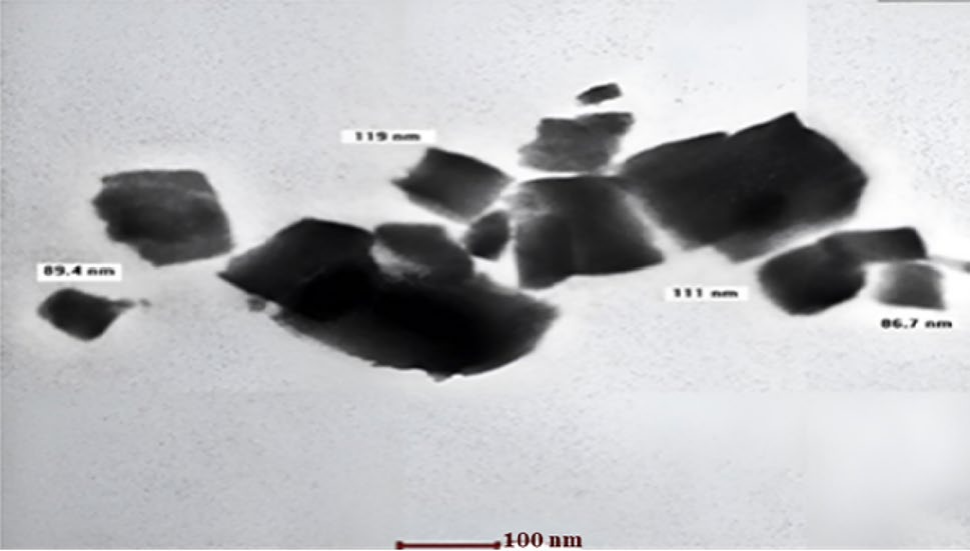
TEM of the cubosomal dispersion.

The morphological appearence of the created cubosomal dispersions was studied using TEM. The photomicrograph obtained is shown in Fig. 1. The particles were cubic in shape and well isolated from one another.

#### In-vitro drug release

**Figure 2:**
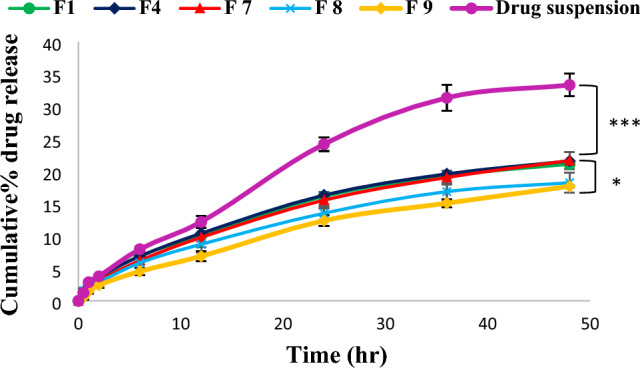
Release profiles of Sim from different cubic formulations and drug suspension, ((***) significance level between F7 and drug suspension, (*) significance level between F7 and F9). One way ANOVA was used for statistical analysis; *** (*p* < 0.001), * (*p* < 0.05).

Figure 2 shows Sim^’^release pattern from drug suspension and different cubic nanoparticle formulations. Slower release (^***^p < 0.001) of Sim from cubosomes was observed compared to its release from drug suspension, this indicated the effect of cubic nano-system in retarding drug release. Moreover, slower (^*^p < 0.05) release of Sim from cubosomes with higher GMO percent compared to those with lower GMO percent was observed; The percent of drug released from F7 (2%GMO) and F9 (6% GMO) after 24 h was 16.12 ± 1.11% and 12.38 ± 0.83% respectively (Fig. 2). These results are due to the unique cubic nanoparticle structure that incorporates lipophilic medicines in the 3-D network of lipid bilayers, which grow in number as the concentration of GMO rises. So more barrier against drug release^40,43, 44^. However, there was no significant effect of tested polymer concentrations (F1 (0.5% F_127_, 2% GMO), F4 (1% F_127_, 2% GMO) and F7 (1.5% F_127_, 2% GMO) on drug release, as the percent of drug released was 21.58 ± 0.04% over 48 h (Fig. 2).

**Figure 3:**
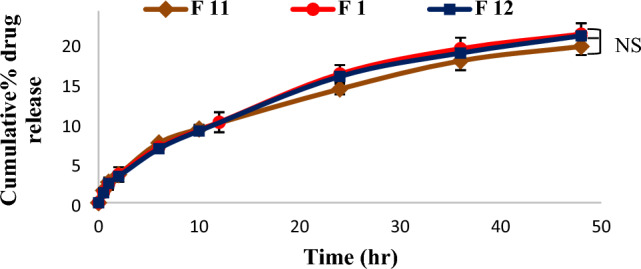
Release profiles of Sim from cubic formulations with different drug concentrations, (NS; non significance between F1, F11 and F12). One way ANOVA was used for statistical analysis; NS (non-significant).

Also, it was found that the studied drug concentrations (F11 (0.35%), F1 (0.25%) and F12 (0.15%)) had no significant effect on drug release as the percent of drug released after 48 h was 20.88 ± 0.47% (Fig. 3).

##### Mechanisms and kinetics of drug release

The mechanism of drug release was detected using different kinetic equations (zero order, first order, Higuchi model). It was found that the release behavior of simvastatin from cubic nanoparticles followed Higuchi’s diffusion model which gave the highest regression coefficient value (r^2^) compared to other release models (Table 3). Equation of Higuchi’s model is described as the following:

$${\mathbf{m}}_{0} - {\mathbf{m}}_{{\mathbf{t}}} = \, {\mathbf{k}}_{{\mathbf{2}}} {\mathbf{t}}^{{{\mathbf{1}}/{\mathbf{2}}}}$$

where (m_o_–m_t_) is the fraction of the drug released at time t and k_2_ is the Higuchi constant^45^. So the Korsmeyer–Peppas equation **M**_**t**_**/M**_**∞**_** = K**_**3**_**t**^**n**^ was used to predict the diffusion mechanism by analyzing the diffusion exponent (n) where M_t_ is the amount of drug released at time t, M_∞_ is the amount of drug released at time t_∞_ and K_3_ is a constant that incorporates the structural and geometric properties of dosage form^46^. According to this model, the logarithm of less than the initial 60% of the release data values was fitted versus the log time to get the value of n. When n = 0.45, The diffusion mechanism follows Fick’s law, For n between 0.45 and 0.89; transport is non-Fickian; n = 0.89 corresponds to Case-II transport; and n > 0.89 for Super Case-II transport^47^. In this study, it was found that diffusion mechanism undergoes non-Fickian transport (n between 0.542 and 0.708) as shown in (Table 3) and this might be explained by the cubic phase's aqueous channels' diffusion control mechanism^48^.

**Table 3:** Kinetic models of simvastatin release from different cubosomal formulations.

Formulations code	Zero order (r^2^)	First order (r^2^)	Higuchi model (r^2^)	Korsmeyer–peppas (n)
F 1	0.971	0.976	0.998	0.597
F 4	0.971	0.977	0.998	0.612
F 7	0.980	0.985	0.999	0.622
F 8	0.976	0.980	0.998	0.542
F 9	0.985	0.988	0.997	0.708
F 11	0.969	0.975	0.999	0.549
F 12	0.973	0.978	0.998	0.601

Results of further characterizations of cubosomes (FT-IR, DSC and stability study) are shown in the supplementary file.

### Characterization of simvastatin loaded cubogels

#### Visual appearance and pH of prepared cubogels

The systems developed were white and homogeneous cubogels with no evidence of separation or precipitation, according to visual inspection and acceptable pH values to be applied on the skin were obtained, Supplementary data provide pH values of prepared cubogels.

#### Rheological behavior of prepared cubogels

**Figure 4:**
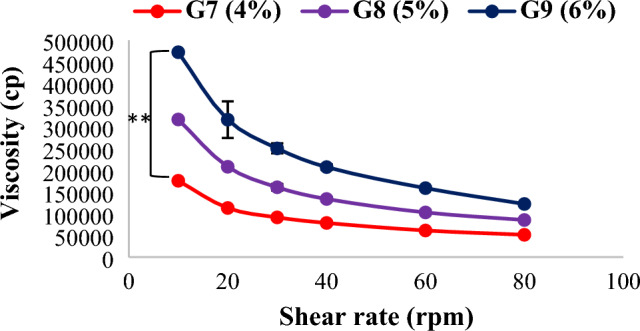
The rheological behavior of HPMC based cubogels, (^(^**^)^ significance level between G7 and G9). One way ANOVA was used for statistical analysis; ** (*p* < 0.01).

The viscosity values would have an impact on the flow characteristics of cubogels when applied on the skin and also affect the behavior of drug release. Rheological profile of HPMC cubogels (Fig. 4) revealed that the formulations contained higher levels of the polymer (HPMC) were more viscous (^**^p < 0.01) than those containing lesser amount of it^49^. These results could be attributable to the polymer network's cross-linking has increased. All cubogel formulations exhibited pseudo-plastic flow, as a reduction in viscosity was obtained upon elevation of the angular velocity. As the molecules orient themselves in the direction of flow, lowering the barrier to movement^50^. Shear thinning characteristic is preferred because topical semisolid formulations should thin during application and thicken otherwise^51^. Similar results were observed with chitosan and carbopol based cubogels, see supplementary data.

#### In-vitro drug release from prepared cubogels

**Figure 5:**
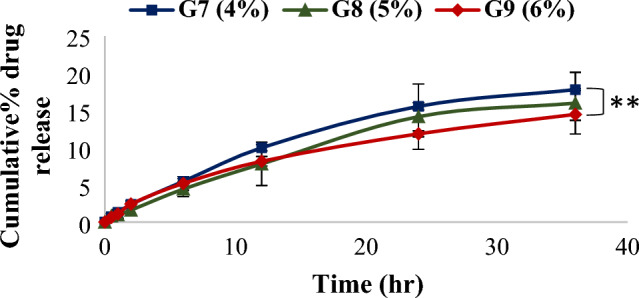
Release profile of simvastatin from HPMC based cubogels, ((**) significance level between G7 and G9). One way ANOVA was used for statistical analysis; ** (*p* < 0.01).

Pattern of drug release from all prepared cubogels with different gelling agents in different concentrations were studied over 36 h period. For HPMC based cubogels, it was found that raising the HPMC concentration from 4% (G7) to 6% (G9) resulted in a considerable reduction in drug release (^**^p < 0.01) from (17.70 ± 0.14%) to (14.40 ± 0.81%) respectively as shown in Fig. 5. These results were in compliance with rheology results as an increase in polymer concentration resulted in higher viscosity of the prepared cubogel due to the gels’polymer chains have high density. As a result, the diffusion pathway's length increased, slowing the rate of drug release^52^. See supplementary data for chitosan and carbopol based cubogels results. The mechanism and behavior of drug release from the prepared cubogels were investigated, the results showed that The Higuchi model has the highest R^2^ value of all the release kinetic models for most cubogels with non-Fickian transport (n between 0.45 and 0.89) as shown in the supplementary file.

#### Effect of adding solubilizers on drug release from different cubogels

**Figure 6:**
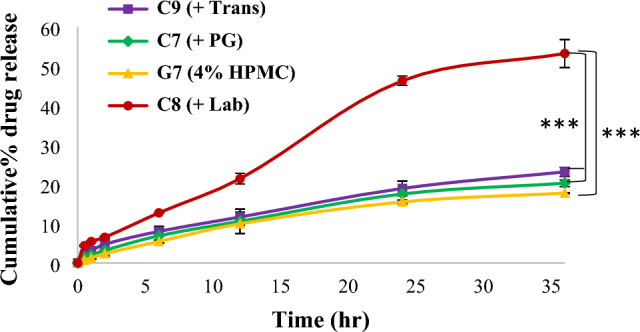
Release profile of simvastatin from HPMC based cubogels with different solubilizers, ((***) significance level between C8 and G7, (***) significance level between C8 and C7, also between C8 and C9). One way ANOVA was used for statistical analysis; *** (*p* < 0.001).

The cubogel formulae (G2 (F1 in 3% chitosan), G4 (F1 in 1% carbopol) and G7 (F1 in 4% HPMC)) were selected based on rheology and in-vitro release results for studying the effect of adding solubilizers on drug release. It was found that significant increase in the amount of drug released from cubogels containing (5%) of transcutol, PG or labrasol ((C1-C9); the composition is shown in the supplementary file) compared to original cubogels (G2, G4, G7). This can be due to enhancing Sim's solubility and hence the gradient of the drug's concentration in solution, facilitating Sim release from the dosage form. For HPMC based cubogels (C7(G7 + 5% PG), C8 (G7 + 5% labrasol) and C9 (G7 + 5% Transcutol)), it was found that the drug release enhanced significantly (^***^p < 0.001) with formulation containing labrasol (C8) compared to other formulations (C7, C9 and G7), as the released drug was 53.32 ± 3.56% after 36 h (Fig. 6). Pearson correlation coefficient between G7 and C7, C8, C9 was calculated to evaluate the effect of studied solubilizers in enhancing drug release from G7 cubogel. The results showed that the values of correlation coefficients between G7 and C7, C8 and C9 were 0.998, 0.986 and 0.994 respectively, which indicated the strong positive correlation among the variables^32^. (Further results and Sim release kinetics from studied cubogels after adding solubilizers are shown in the supplementary file).

#### Ex-vivo permeation study

**Figure 7:**
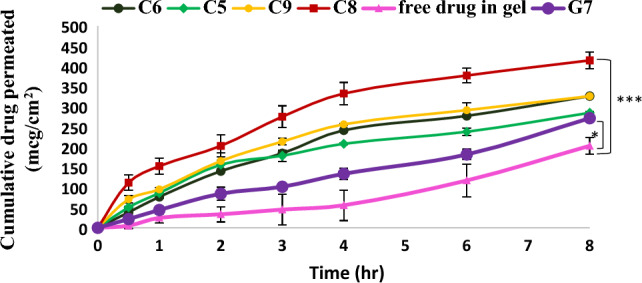
Ex-vivo permeation profiles of simvastatin from different cubogels against free drug gel, ((***) significance level between C8 and free drug in gel, (*) significance level between G7 and free drug in gel). One way ANOVA was used for statistical analysis; *** (*p* < 0.001), *(*p* < 0.05).

Skin^’^permeability of selected formulations; C5(G4 + 5% lab), C6 (G4 + 5% Trans), C8 (G7 + 5% lab), C9 (G7 + 5% Trans) and G7 (F1 + 4% HPMC) were studied and free drug gel preparation was used as a control. Figure 7 shows the commulative amount permeated of Sim through rat ^‘^skin from various formulations as a function of time. The influence of cubosomal nano-system on skin permeation of Sim was evaluated through studying ex-vivo permeation for G7 against free drug in gel. It was found that significant(^*^p < 0.05) increase in amount of Sim permeated from G7 (271.57 ± 7.31 mcg cm^−2^) compared to that permeated from free drug in gel preparation (218.00 ± 21.00 mcg cm^−2^) after 8 h. This improvement in drug permeation through skin is attributed to the similarity of cubic phase structure of cubosomes to stratum corneum so cubosomes showed penetration enhancing impact on the skin as the lipid component of the nanoparticles combined with the lipids of the stratum corneum and consequently fluidized the stratum corneum^53^. For other cubogel formulations (C5, C6, C8 and C9), the amount of drug permeated after 8 h was higher than that permeated from free drug preparation and G7 (Fig. 7). This can be explained to be due to the synergetic effect of cubosomal nano-system and the presence of solubilizers (labrasol, Transcutol) in these preparations which reduced lipid bilayer barrier resistance by modifying intercellular stratum corneum domains, acting as skin penetration enhancers^54^, so significant enhancement in skin permeation of Sim was obtained. Sim penetration into rat skin was measured, and the amount (Q) was plotted against time. The slope of the linear portion of the figure was used to compute the transdermal drug flux (Jss) and the apparent permeability coefficient (P_app_) was determined through **Jss/C**_**o**_ equation where C_o_ is the initial drug concentration^55^. The results showed that HPMC based cubogel (C8) achieved a considerable (^***^p < 0.001) increase in skin permeation effect compared to free drug in gel preparation (Table 4), resulting in approximately two-fold (1.78 fold) increase in skin permeation of Sim and also higher penetration ability compared to all other cubogel formulations so it was selected for in-vivo study.

**Table 4:** Ex-vivo permeation parameters of simvastatin from different formulations through rat dorsal skin.

Parameter	Q (mcg cm^−2^)	Jss (mcg cm^−2^ h^−1^)	P_app_x10^3^ (cm/h)
Formulation code			
C5	284.88 ± 3.18	32.88 ± 0.55	13.15 ± 0.00
C6	326.30 ± 0.47	40.48 ± 0.24	16.19 ± 0.00
C8	386.18 ± 20.20	46.18 ± 2.12	18.47 ± 0.00
C9	326.12 ± 2.70	40.37 ± 4.03	16.14 ± 0.00
G7	271.57 ± 7.31	31.82 ± 0.77	12.72 ± 0.00
Free drug gel	218.00 ± 21.00	25.92 ± 3.45	10.36 ± 0.00

### In vivo wound healing activity

#### Wound closure

**Figure 8:**
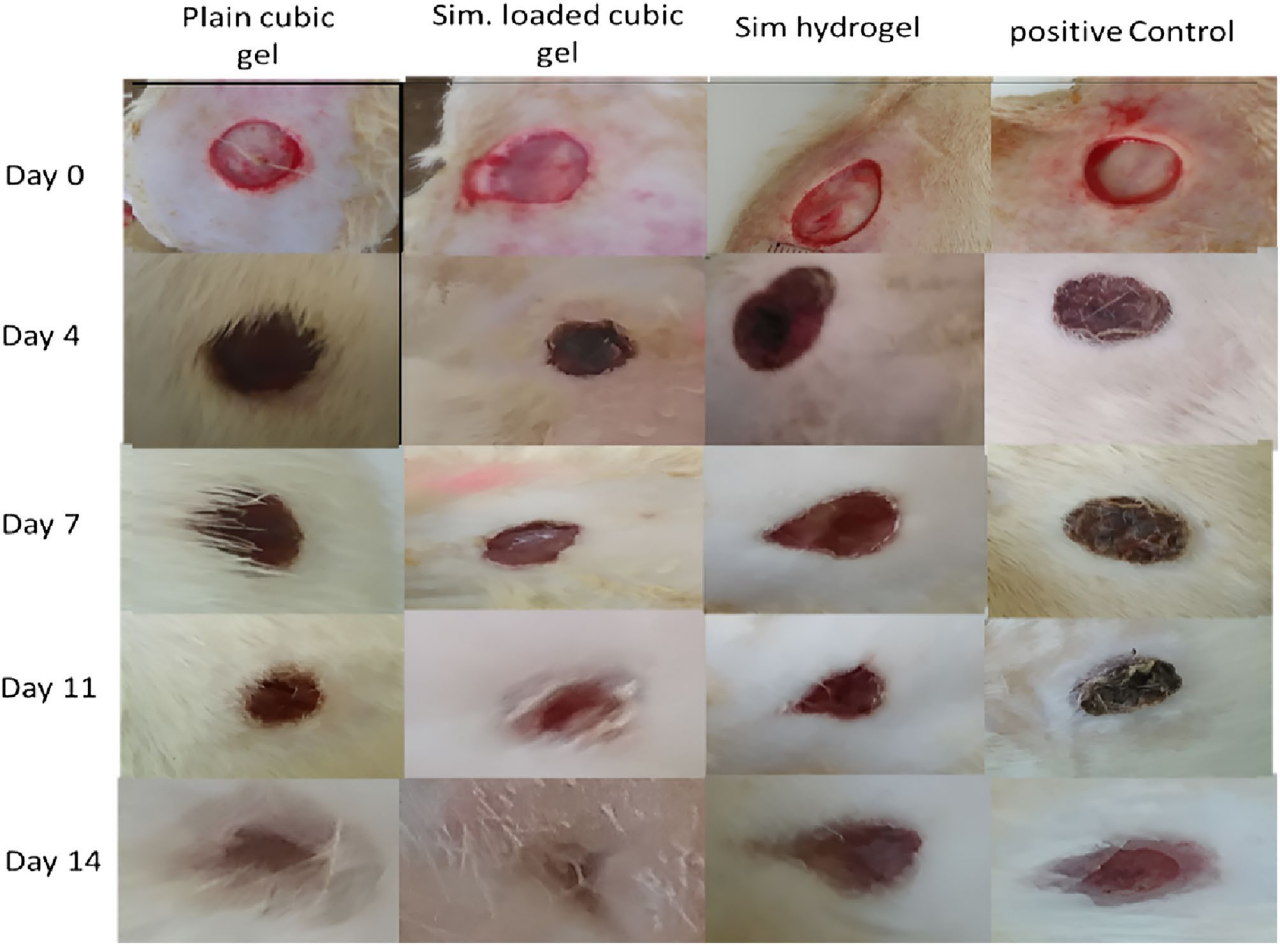
Wound healing progression in rats of different experimental groups 4,7, 11 and 14 days post-wounding.

**Figure 9:**
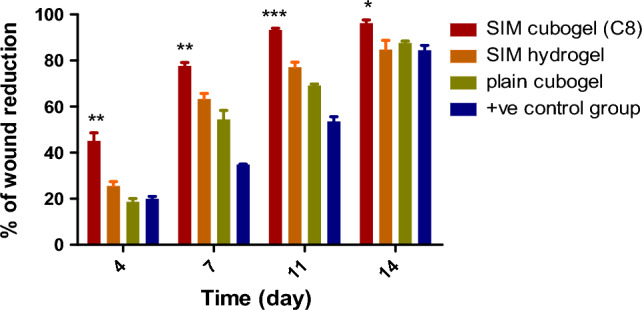
Relative reduction in wound area in rats of different experimental groups (simvastatin cubogel (C8) group, plain cubogel group, simvastatin hydrogel group and + ve control group) at 4, 7, 11 and 14 days post-wounding. ((**) significance level between Sim cubogel (C8) group and other groups at 4, 7 days, (***) significance level between Sim cubogel (C8) group and other groups at 11 days, (*) significance level between Sim cubogel (C8) group and other groups at 14 days). One way ANOVA was used for statistical analysis; *** (*p* < 0.001), **(*p* < 0.01),* (*p* < 0.05).

Figure 8 depicts the wound healing progression for rat groups at 4,7, 11, and 14 days after wounding, whereas Fig. 9 depicts the relative reduction in wound size for the groups. The results showed that the group received Sim loaded cubogel has the highest percentage in wound closure at all days compared to other treated groups. The Sim loaded cubogel group(C8) had a substantial (^***^p < 0.001) higher wound closure percent (92.32 ± 1.61%) than the plain Cubogel group (69.15 ± 0.63%) after 11 days (Fig. 9), which approves the effect of simvastatin as a wound healing agent. The wound reduction in the free Sim hydrogel group was slower (^***^p < 0.001) than in the Sim loaded cubogel group with percent of closure 77.00 ± 2.26% after 11 days (Fig. 9). This can be explained by the higher amount of medication delivered to the skin via the cubosomal nano-system which was in compliance with ex-vivo permeation results. Also, the higher reduction (^***^p < 0.001) in wound area for Sim loaded cubogel compared to positive control group which had wound reduction only 52.07 ± 0.00% after 11 days, indicated the successful effect of Sim loaded cubosomal nano-system in acceleration of wound healing process. On the other hand, the wound closure percents for Sim hydrogel, plain cubogel and positive control groups were not statistically different until the seventh day. On 7 and 11 days, the order in wound healing progression was Sim hydrogel group > plain cubogel group > positive control group, whereas at day 14 all wounds were closed non significantly.

#### Histopathological study

Wound healing is a complex repairing process which controlled by several pathways to keep skin integrity. One of the most crucial factors in the processing of wound closure that identifies the efficiency of wound healing is re-epithelialization. The re-epithelialization phase, which typically follows and overlaps with the inflammatory phase, is characterized by epithelial proliferation and migration across the provisional matrix within the wound. As a result, it promotes capillary expansion, collagen synthesis, and the development of granulation tissue at the site of damage^56,57^. Histological examination was conducted to investigate the healing progression. The findings demonstrated that rats treated with Sim-loaded cubosomal gel had newly formed blood vessels and granulation tissue formulation, indicating a successful re-epithelialization process in the skin. As a result, this group of rats showed more rapid wound healing than other rat groups.

**Figure 10:**
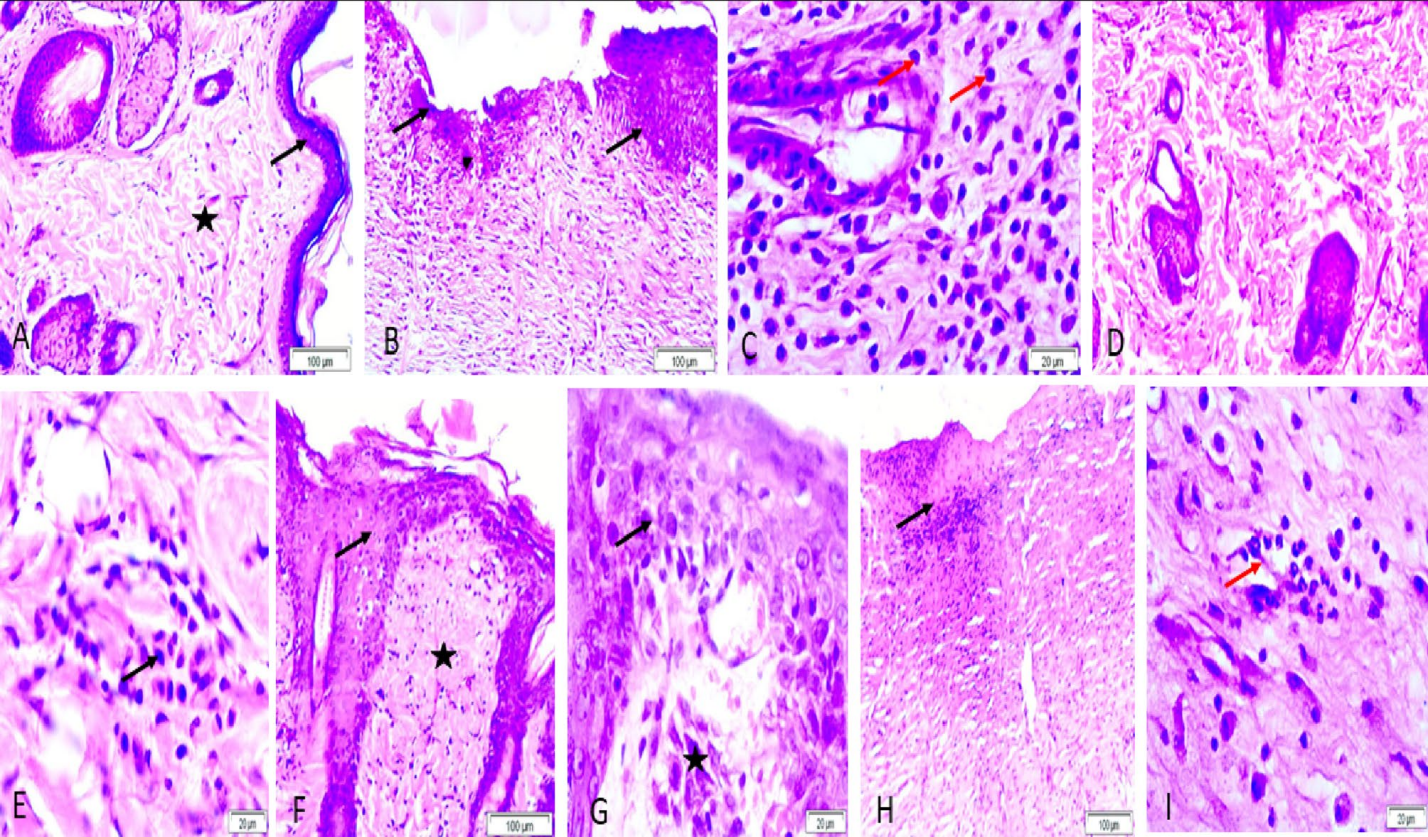
Representative micrograph of rat skin with H&E stain: (**A**) healthy rats group: arrow(epidermis)and star (dermis), (**B**, **C**) (+ ve control group): black arrows (necrosis and sloughing of the epidermis), white arrow(with inflammatory cells especially macrophage) and red arrows (lymphocytes), (**D**, **E**) plain cubogel group: arrow (inflammatory cells infiltrated in the dermis), (**F**, **G**) Sim loaded cubogel group: arrow(normal epidermis), star (normal dermis) and (**H**, **I**) Sim hydrogel group: black arrows(necrosis and sloughing of the epidermis) and red arrows(infiltration of the dermis with inflammatory cells).

Figure 10 illustrates the histological microscopic differences for normal rat skin and for wounded tissues; (A) healthy rats group (-ve control) which provides the basic structure of normal skin with normal epidermis and dermis; (B, C) rats subjected to experimental wound with no treatment (+ ve control) which showed necrosis and sloughing of the epidermis, congestion, and heavy infiltration of the dermis with inflammatory cells, particularly macrophages and lymphocytes, (D, E) wounded rats that received plain cubogel and revealed slightly normal epidermis and dermis with inflammatory cells infiltrated in the dermis; (F, G) rats which treated with the selected Sim loaded cubogel (C8) that showed higher rate of re-epithelialization with normal epidermis and dermis after the period of treatment compared to all other wounded groups. (H, I) refers to that group treated with Sim hydrogel which resulted in necrosis, sloughing of the epidermis and infiltration of the dermis with inflammatory cells. Based on these results Sim loaded cubosomal gel can be considered a successful approach for healing of wounds.

## Conclusion

In the current work, cubosomes were successfully prepared with favourable physicochemical properties and simvastatin was efficiently entrapped in cubosomal nanoparticles with EE % of 93.95 ± 0.49 %. Various cubogels were created using the chosen cubosomal formulation (F1). From these cubogels, HPMC based cubogel (C8) was found to have superior characters for topical administration, as well as improved permeability of simvastatin through skin compared to free drug hydrogel. This was attributed to the penetrating boosting action of cubosomes and other penetrating enhancers. Finally, The efficacy of simvastatin in accelerating wound healing was evaluated by histopathological evaluation. Based on these results, cubic nanoparticles are thought to be a promising tool for delivering simvastatin to the skin layers. In the future, we need to concentrate on the preparation of simvastatin cubosomes precursor-microparticles (CPMs), which will be spray dried from aqua-free precursor solutions to improve simvastatin cubosomes efficacy and stability during storage.

### Supplementary Information

Supplementary Information.

## Data Availability

The datasets analysed during the current study are available from the corresponding author on reasonable request.
