# Supplementary material for: Formulation and evaluation of simvastatin cubosomal nanoparticles for assessing its wound healing effect
Source: Sci Rep. 2023 Oct 20;13:17941. doi: 10.1038/s41598-023-44304-2 (PMC10589326; doi:10.1038/s41598-023-44304-2)
Supplement: Supplementary file 1 — Supplementary Information. [file 41598_2023_44304_MOESM1_ESM.pdf]

Supplementary information for:

Formulation and evaluation of simvastatin cubosomal nanoparticles for assessing its wound healing effect

Lamiaa M. Ahmed[1], Khaled M. A. Hassanein[2], Fergany A Mohamed[1], and Tahani H. Elfaham[1*]

[1]Department of pharmaceutics, Faculty of pharmacy, Assiut university, Assiut, Egypt.

[2]Department of Pathology and Clinical Pathology, Faculty of Veterinary Medicine, Assiut University, Assiut 71526, Egypt.

Lamiaa M. Ahmed: lamiamahmoud@pharm.aun.edu.eg; Khaled M. A. Hassanein: Khaledhassanein@aun.edu.eg; Fergany A Mohamed: fergmed@yahoo.com ; Tahani H. Elfaham: Telfaham@aun.edu.eg

[*]Corresponding author at: Department of Pharmaceutics, Faculty of Pharmacy, Assiut University, Assiut 71526, Egypt. Telfaham@aun.edu.eg

Table (S1): Composition of the prepared cubogels

Formulation code	Gelling agent		
	HMW Chitosan (Cs)	Carbopol (CP) 934	HPMC
G1	3% (w/v)	-	-
G2	3.5 % (w/v)	-	-
G3	4 % (w/v)	-	-
G4	-	1% (w/v)	-
G5	-	2% (w/v)	-
G6	-	3% (w/v)	-
G7	-	-	4% (w/v)
G8	-	-	5% (w/v)
G9	-	-	6% (w/v)

Table (S2): Composition of the prepared cubogels with different solubilizers

Formulation code	Cubogel code	Propylene glycol (PG) (w/v)	Labrasol (lab) (w/v)	Transcutol (Trans)(w/v)
C1	G2	5%	-	-
C2	G2	-	5%	-
C3	G2	-	-	5%
C4	G4	5%	-	-
C5	G4	-	5%	-
C6	G4	-	-	5%
C7	G7	5%	-	-
C8	G7	-	5% (w/v)	-
C9	G7	-	-	5% (w/v)

Table (S3) Mean particle size, PDI and zeta potential results after storage of cubic nanoparticles (F1) at different temperatures:

Sampling Time	Particle size(nm)		PDI		Zeta potential	
	at 4 ℃	at 25 ℃	at 4 ℃	at 25 ℃	at 4 ℃	at 25 ℃
Initial	114 ± 0.71	114.00 ± 0.71	0.14 ± 0.01	0.14 ± 0.01	-20.95 ± 5.30	-20.95 ± 5.30
2 weeks	125.10 ±10.32	121.80 ± 9.66	0.179 ± 0.05	0.22 ± 0.06	-16.10 ± 2.43	-18.90 ± 1.77
1 month	124.00 ± 12.58	122.20 ± 12.50	0.165 ± 0.10	0.21 ± 0.03	-21.00 ± 0.85	-19.00 ± 0.57
2 months	119.10 ± 1.69	115.20 ± 2.69	0.12 ± 0.08	0.16 ± 0.01	-28.20 ± 0.28	-20.60 ± 1.70
3 months	118.30 ± 6.36	121.50 ± 4.10	0.18 ± 0.09	0.20 ± 0.04	-21.20 ± 5.52	-16.10 ± 1.13
4 months	115.15 ± 2.61	114.55 ± 7.71	0.19 ± 0.08	0.19 ± 0.04	-23.10 ± 6.93	-23.10 ± 2.83
5 months	119.6 ± 0.282	120.45 ± 7.42	0.18 ± 0.06	0.18 ± 0.01	-23.25 ± 2.33	-13.40 ± 0.14
6 months	119.5 ± 0.424	160.70 ± 64.49	0.18 ± 0.07	0.39 ± 0.26	-27.95 ± 0.35	-21.85 ± 1.63

Table (S4): pH values for prepared cubogels

Cubogel Code	pH value	Cubogel Code	pH value	Cubogel Code	pH value
**G1**	4.78 ± 0.16	**G4**	6.63 ± 0.48	**G7**	4.75 ± 0.12
**G2**	4.70 ± 0.06	**G5**	5.63 ± 0.04	**G8**	4.95 ± 0.18
**G3**	4.74 ± 0.01	**G6**	4.98 ± 0.00	**G9**	5.02 ± 0.00

Table (S5): Kinetic models of simvastatin release from different cubogel formulations

Formulation code	**Zero order (r^2)**	**First order (r^2)**	**Higuchi model (r^2**	**Korsmeyer-peppas (n)**
G1	0.997	**0.997**	0.981	0.760
G2	0.995	**0.996**	0.984	1.01
G3	0.975	0.979	**0.997**	0.546
G4	0.988	0.992	**0.997**	0.659
G5	0.981	0.985	**0.997**	0.663
G6	0.986	0.989	**0.996**	0.612
G7	0.974	0.978	**0.996**	0.748
G8	0.980	0.983	**0.992**	0.752
G9	0.973	0.978	**0.999**	0.715

Table (S6): Kinetic models of simvastatin release from different cubogels containing solubilizers

Formulation code	Zero order (r^2)	First order (r^2)	Higuchi model (r^2	Korsmeyer-peppas (n)
C1	0.967	0.974	**0.995**	0.383
C2	0.987	**0.990**	0.988	0.587
C3	0.980	0.985	**0.994**	0.443
C4	0.982	0.986	**0.987**	0.554
C5	0.995	**0.999**	0.991	0.574
C6	0.995	**0.100**	0.993	0.582
C7	0.980	0.984	**0.997**	0.631
C8	0.987	**0.990**	0.979	0.629
C9	0.987	0.992	**0.997**	0.558

Table(S7): pH values of selected simvastatin loaded cubic gel (C8) stored at 4-8℃ and 25±2℃.

**Sampling Time**	**pH**	
	**at 4 ℃**	**at 25 ℃**
**Initial**	4.83 ± 0.33	4.83 ± 0.33
**2 weeks**	4.45 ± 0.04	4.27 ± 0.15
**1 month**	4.19 ± 0.02	3.67 ± 0.18
**2 months**	4.46 ± 0.38	3.38 ± 0.17
**3 months**	3.52 ± 0.03	3.17 ± 0.04

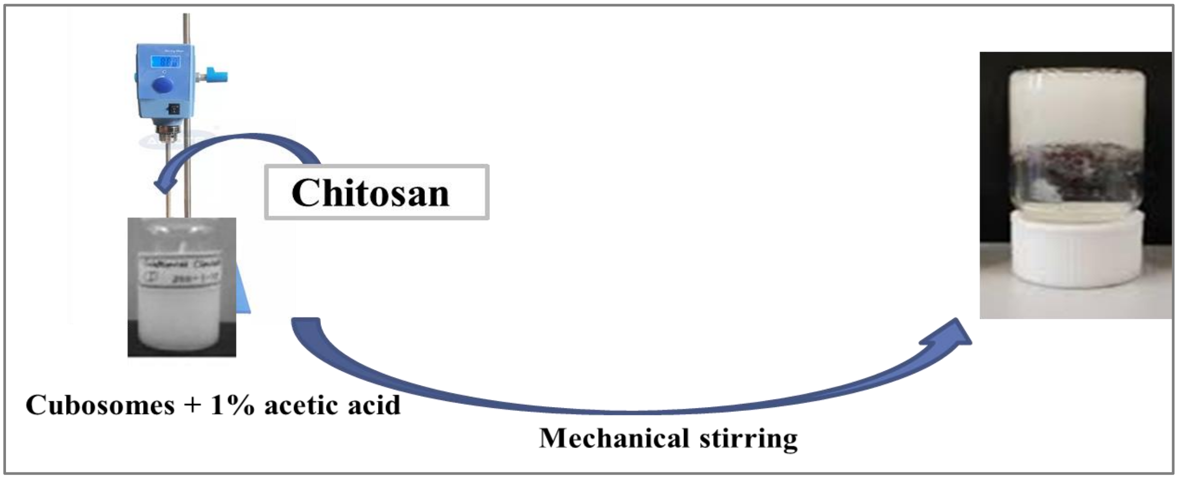

Figure (S1): preparation of chitosan (Cs) based cubogels. Formulations were prepared with different chitosan concentrations (G1 (3% w/v), G2 (3.5% w/v) and G3 (4% w/v)).

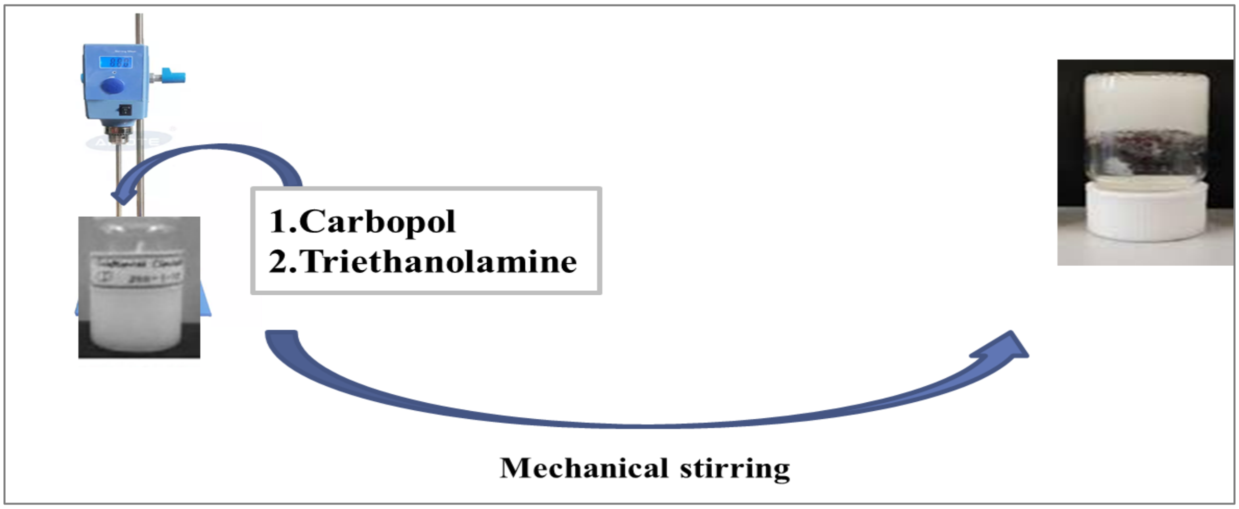

Figure (S2): preparation of carbopol (CP) based cubogels. Formulations were prepared with different carbopol concentrations (G4 (1% w/v), G5 (2% w/v) and G6 (3% w/v)).

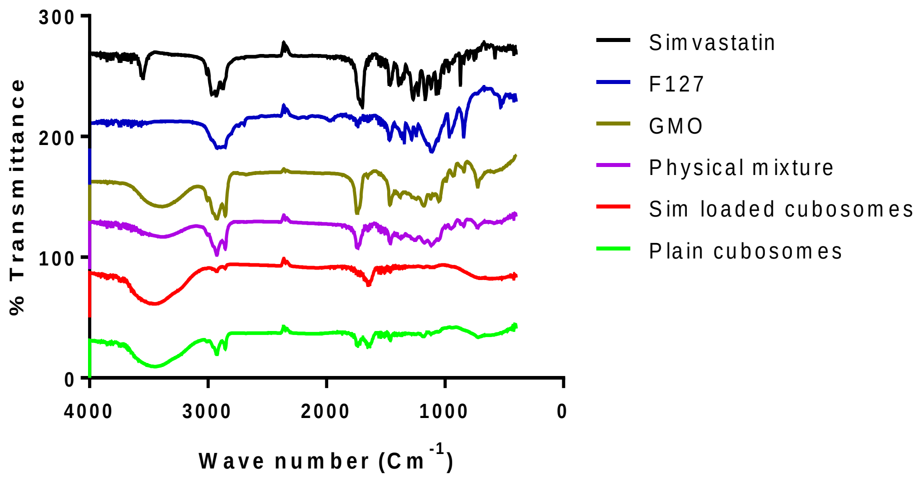

Figure(S3): Comparative FTIR spectra of simvastatin, F_{127},GMO, physical mixture, plain and Sim loaded cubosomal dispersions.

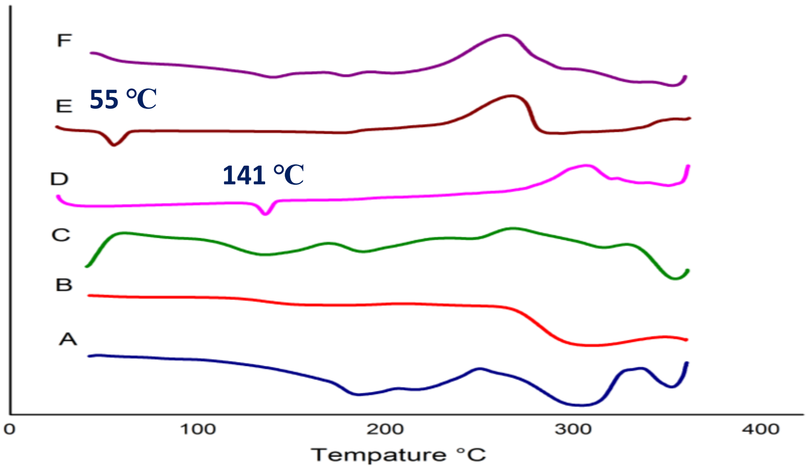

Figure (S4): DSC thermograms of A):plain cubosomes, B) Sim loaded cubosomes, C) physical mixture , D) simvastatin, E) F_{127} and F) GMO.

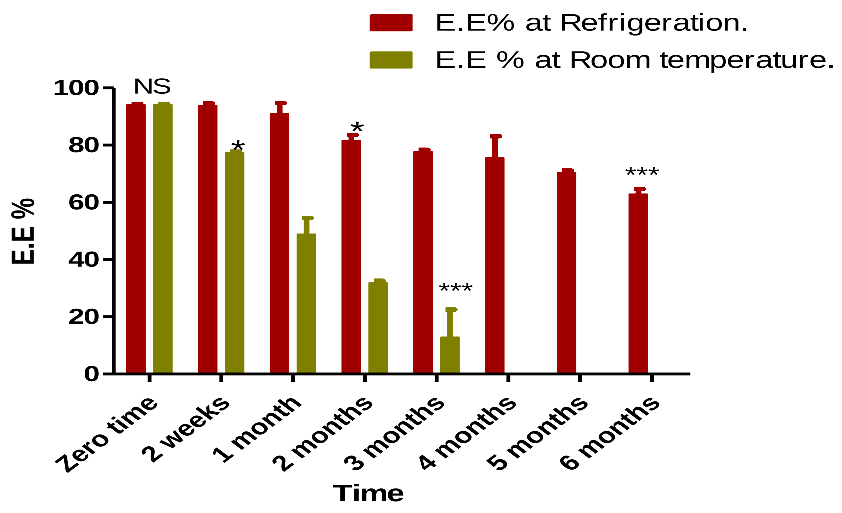

Figure (S5): E.E % results of simvastatin loaded cubic nanoparticles stored (F1) at 4-8℃ and 25±2℃ at various time intervals. One way ANOVA was used for statistical analysis; NS (non-significant), * (P<0.05) and *** (P< 0.001), each time interval was compared to zero time.

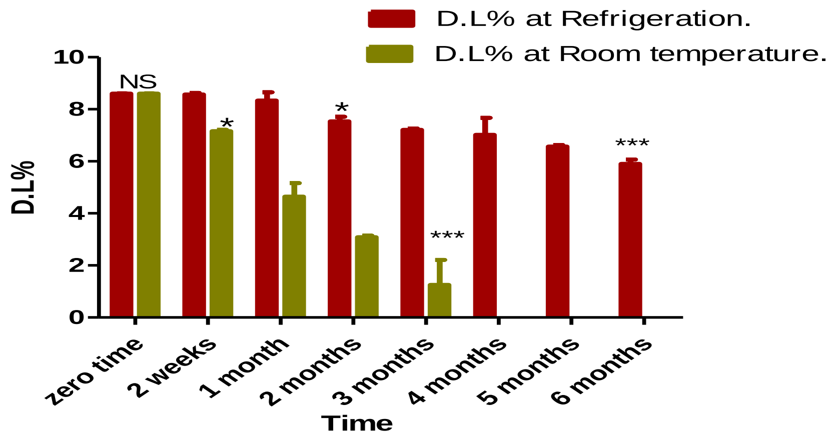

Figure(S6) : D.L % results of simvastatin loaded cubic nanoparticles (F1) stored at 4-8℃ and 25±2℃ at various time intervals. One way ANOVA was used for statistical analysis; NS (non-significant),* (P<0.05) and *** (P< 0.001), each time interval was compared to zero time.

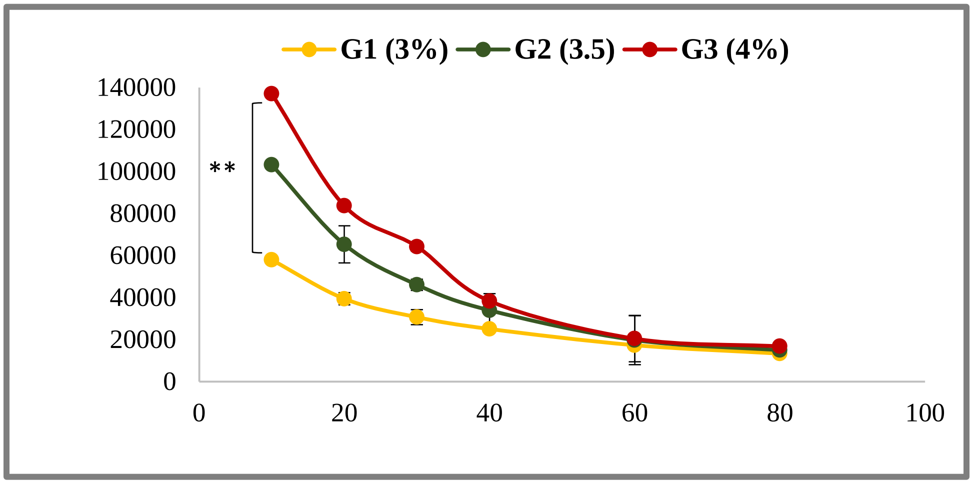

Figure (S7): The rheological behavior of chitosan based cubogels. One way ANOVA was used for statistical analysis; ** (P<0.01).

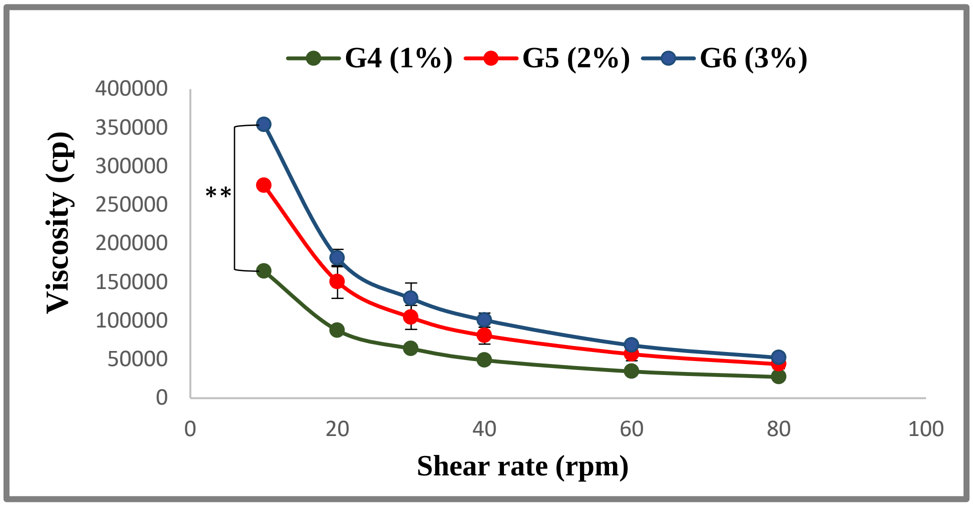

Figure (S8): The rheological behavior of carbopol based cubogels. One way ANOVA was used for statistical analysis; ** (P<0.01).

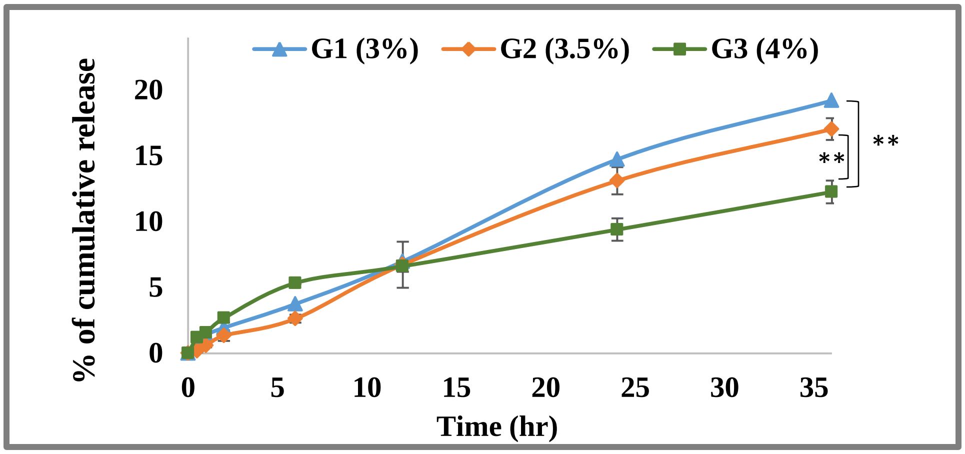

Figure (S9): Release profile of simvastatin from chitosan based cubogels. One way ANOVA was used for statistical analysis; ** (P<0.01).

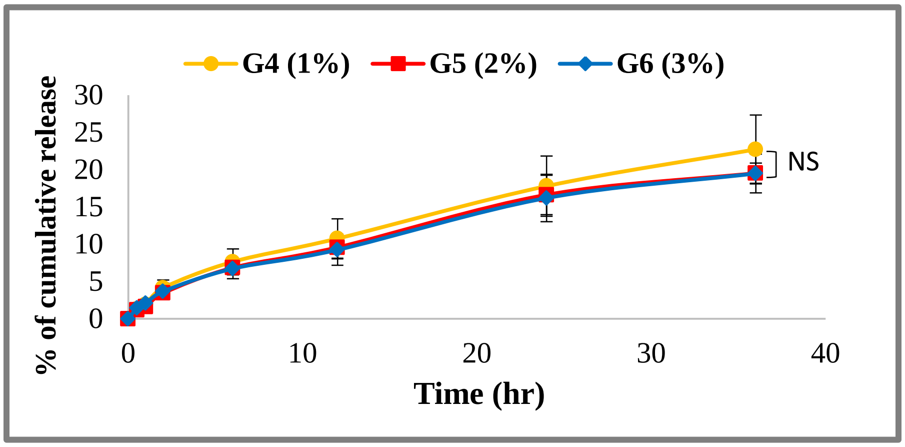

Figure (S10): Release profile of simvastatin from carbopol based cubogels. One way ANOVA was used for statistical analysis; NS (non-significant).

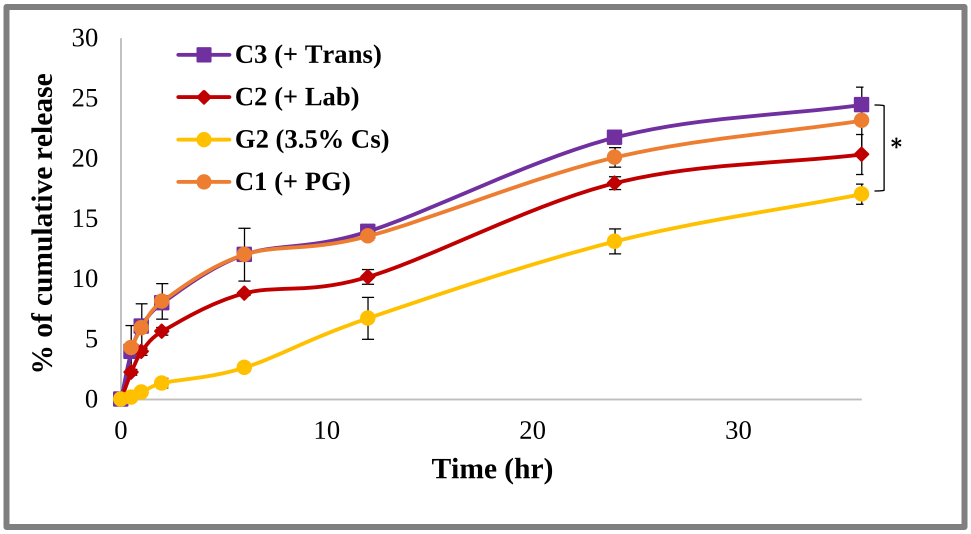

Figure (S11): Release profile of simvastatin from chitosan based cubogels with different solubilizers. One way ANOVA was used for statistical analysis; * (P<0.05).

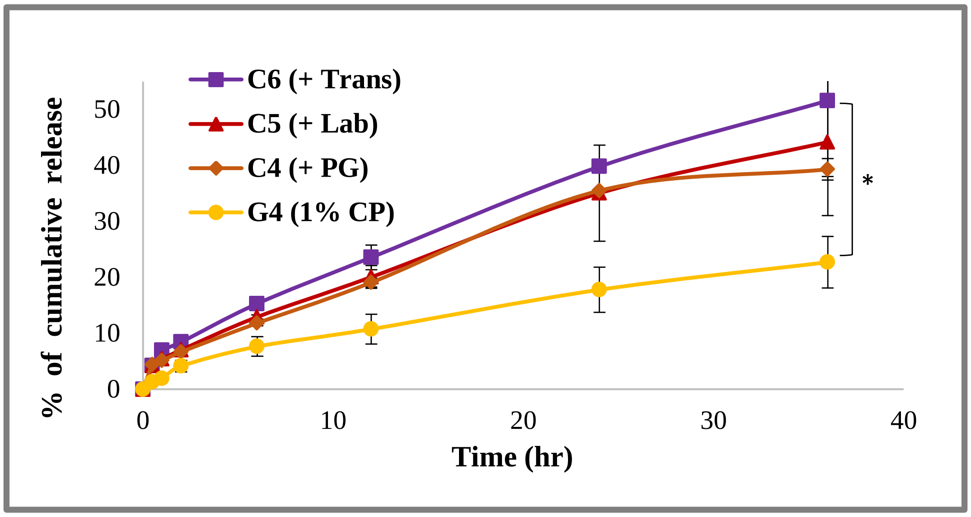

Figure (S12): Release profile of simvastatin from carbopol based cubogels with different solubilizers. One way ANOVA was used for statistical analysis; * (P<0.05).

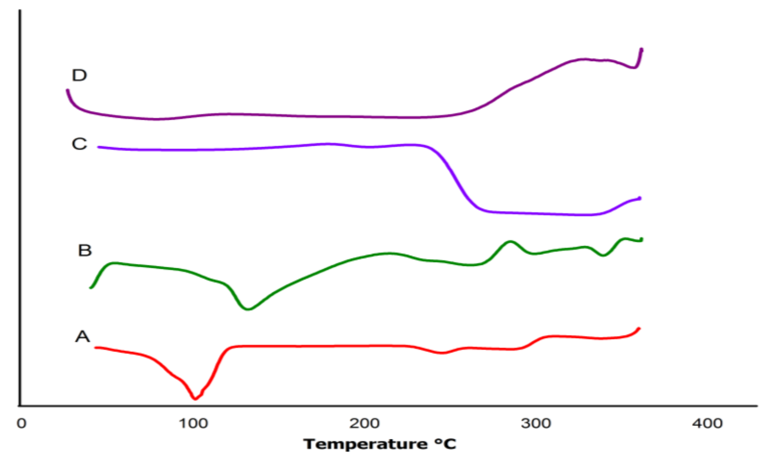

Figure(S13): DSC thermograms of A)selected cubogel (C8), B) physical mixture, C)Labrasol, D) HPMC.

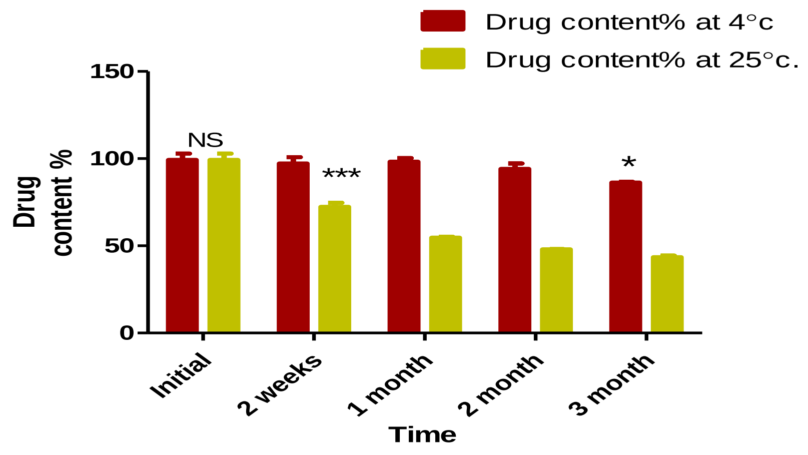

Figure (S14): Sim content (%) results of selected simvastatin loaded cubic gel(C8) stored at 4-8℃ and 25±2℃ for 3 months. One way ANOVA was used for statistical analysis; *** (P<0.001), *(P<0.05), NS (non-significant), all compared to zero time.

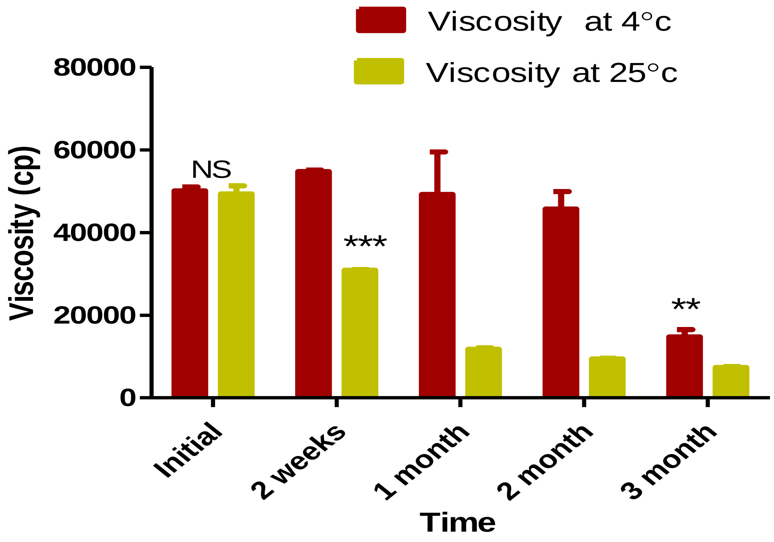

Figure (S15): Viscosity values of selected simvastatin loaded cubic gel (C8) stored at 4-8℃ and 25±2℃ for 3 months. One way ANOVA was used for statistical analysis; *** (P<0.001), **(P<0.01), NS (non-significant), all compared to zero time.

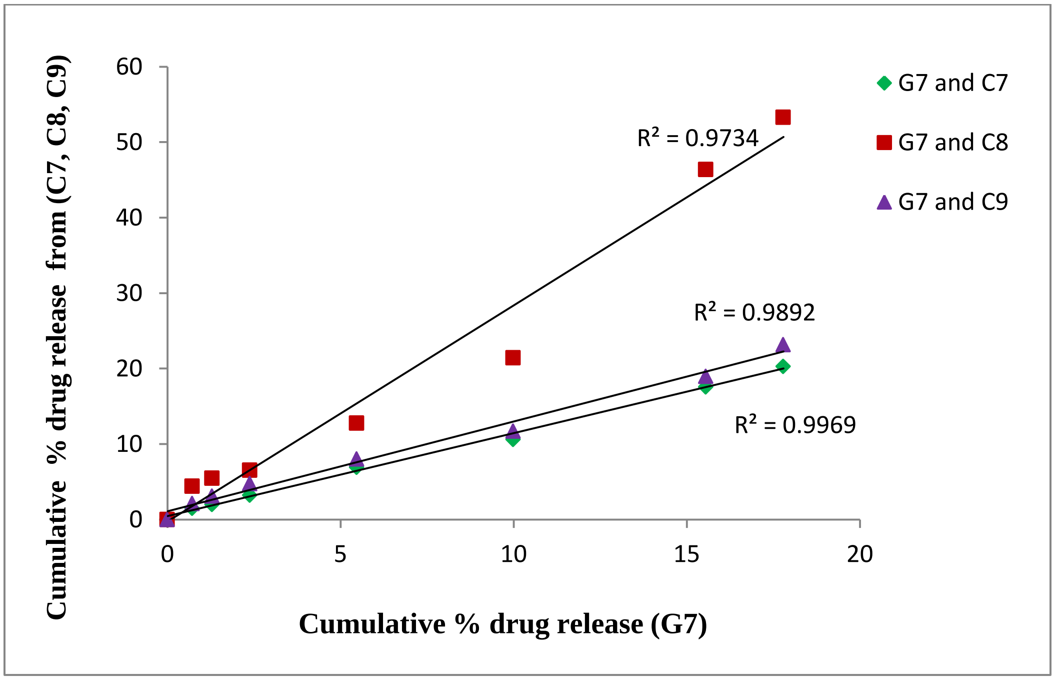

Figure (S16): Pearson correlation coefficients between cubogel G7 (F1 in 4% HPMC) and the cubogel with different solubilizers, C7 (G7+5% PG), C8 (G7+5% labrasol) and C9 (G7+5% Transcutol). The figure showed strong positive correlation between cubogel G7 and each solubilizer added.

Fourier transform-infrared spectroscopy (FT-IR):

The FTIR spectra of Sim, GMO, F_{127}, physical mixture of components, plain and Sim loaded cubosomal dispersions were shown in Figure(3) . The characteristic peaks of Sim were presented as; 3553 cm^{-1} (Free O–H stretching vibration), 2959, 2872 cm^{-1} (C–H stretching vibrations) and sharp peak at 1714 cm^{-1} (stretching vibration of ester and lactone carbonyl functional group). The FTIR spectrum of physical mixture was revealed that, there were no significant changes in Sim peaks. As a result, Sim is compatible with the nano-carrier ingredients. Based on Sim loaded cubosomal dispersion spectrum, significant difference in Sim characteristic peaks that can be attributed to its incorporation in the nano system with amorphous state. The main peaks of F_{127} were presented as 2891 cm^{-1} (C—H stretch), 1344 cm^{-1} (O—H stretch) and 1120 cm^{-1} (C—O stretch. Whereas characteristic bands of GMO were observed at 3473 cm^{-1} (–OH stretching), 2940 cm^{-1} (CH_2 stretching) and 1734 cm^{-1} (C = O ester).

Differential scanning calorimetric (DSC) analysis:

The resulting DSC thermograms of Sim, GMO, F_{127}, physical mixture, plain cubosomes and Sim loaded cubosomes are shown in Figure (4). The thermogram of GMO having no strong endothermic peak, while the thermogram of F_{127} having a distinctive endothermic peak at 55.5°C due to its melting. The disappearance of the F_{127} characteristic peak from the plain cubosomal thermogram (A), implies that it is involved in the creation of the nano-system and interacted with it. There were observed a prominent endothermic peak for Sim at 141 °C, indicating its melting point. The DSC thermogram of the physical mixture demonstrated that there is no interaction between Sim and other excipients, due to the persistence of the drug endothermic peak with observed broadening that can be justified to be due to moisture impact. However, in the thermogram of Sim loaded cubosomes, the melting point endothermic peak of Sim was completely absent, indicating that the drug's incorporation in the cubosomal system was not in a crystalline state.

Differential scanning calorimetric (DSC) analysis for cubogel:

Figure (10) shows the DSC thermograms of (A) selected cubogel(**C8** (G7+5% labrasol), (B) physical mixture of cubogel components, (C) labrasol, (D) HPMC. The DSC thermogram of physical mixture showed an endothermic peak at 140 °C, which is characteristic of Sim due to its melting that suggests that Sim and other gel components are compatible. HPMC and labrasol thermograms exhibit no abrupt endothermic peaks.
